# Different profiles of acute graft pyelonephritis among kidney recipients from standard or elderly donors

**DOI:** 10.3389/fmed.2024.1342992

**Published:** 2024-05-14

**Authors:** Rita Tarragoni, Giovanni Congiu, Alberto Mella, Giovanni Augelli, Fabrizio Fop, Caterina Dolla, Ester Gallo, Maria Cristina Di Vico, Riccardo Faletti, Andrea Bosio, Paolo Gontero, Cristina Costa, Rossana Cavallo, Filippo Mariano, Silvia Corcione, Francesco Giuseppe De Rosa, Paolo Fonio, Luigi Biancone

**Affiliations:** ^1^Renal Transplantation Center “A. Vercellone,” Division of Nephrology Dialysis and Transplantation, Department of Medical Sciences, Città Della Salute e Della Scienza Hospital and University of Turin, Turin, Italy; ^2^Radiology Unit, Department of Surgical Sciences, University of Turin, Turin, Italy; ^3^Division of Urology, Department of Surgical Sciences, Torino School of Medicine, AOU Città Della Salute e Della Scienza, Turin, Italy; ^4^Microbiology and Virology Unit, University of Turin, Turin, Italy; ^5^Department of Medical Sciences, Infectious Diseases, AOU Città Della Salute e Della Scienza, University of Turin, Turin, Italy

**Keywords:** acute pyelonephritis, kidney transplantation, urinary tract infections, multidrug resistant pathogens, ureteral stenosis

## Abstract

**Background:**

Acute graft pyelonephritis (AGPN) is a relatively common complication in kidney transplants (KTs); however, the effects on allograft function, diagnostic criteria, and risk factors are not well established.

**Methods:**

Retrospective analysis of all consecutive adult KTs was performed between 01 January 2011 and 31 December 2018 (follow-up ended on 31 December 2019) to examine the association between the diagnosis of AGPN (confirmed with magnetic resonance imaging [MRI]) during the first post-transplantation year and graft outcomes.

**Results:**

Among the 939 consecutive KTs (≈50% with donors ≥60 years), we identified 130 MRI-confirmed AGPN episodes, with a documented association with recurrent and multidrug-resistant bacterial urinary tract infections (UTIs) (*p* < 0.005). Ureteral stenosis was the only risk factor associated with AGPN (OR 2.9 [95% CI, 1.6 to 5.2]). KTs with AGPN had a decreased allograft function at the first year (ΔeGFR 6 mL/min/1.73 m^2^ [−2–15] in non-AGPN vs. −0.2 [−6.5–8.5] in AGPN, *p* < 0.001), with similar and negative profiles in KTs from standard or elderly donors. However, only KTs with AGPN and a donor <60 years showed reduced death-censored graft survival (*p* = 0.015); most of this subgroup received anti-thymocyte globulin (ATG) induction (40.4% vs. 17.7%), and their MRI presented either a multifocal AGPN pattern (73.9% vs. 56.7%) or abscedation (28.3% vs. 11.7%). No difference was noted in death-censored graft survival between early (<3 months post-KT) or late (3–12 months) AGPN, solitary/recurrent forms, or types of multidrug-resistant pathogens. Linear regression confirmed the independent role of multifocal pattern, abscedation, ATG induction, and donor age on the eGFR at the first year.

**Conclusion:**

AGPN, influenced by multifocal presentation, ATG induction, donor age, and abscedation, affects kidney function and significantly impacts allograft survival in KTs with donors <60 years.

## 1 Introduction

Infectious complications remain a significant cause of morbidity and mortality in solid organ transplant (SOT) patients (1). Among them, urinary tract infections (UTIs) were common in all SOTs but had the highest incidence in kidney transplanted (KT) patients (2). UTIs may evolve with graft involvement, causing acute graft pyelonephritis (AGPN).

Although AGPN occurs in a significant percentage of KTs worldwide, some concerns have emerged about the definition of AGPN and its potential role in allograft dysfunction (3, 4).

For example, diagnostic criteria for AGPN included only suggestive clinical symptoms and typical laboratory findings without radiological confirmation (5), and differences between early or late occurrences after transplant are a matter of debate (6, 7).

Based on the microbiological viewpoint, Gram-negative bacilli account for more than 70% of UTIs in KTs (8–11). Additionally, many AGPN episodes are caused by multidrug resistant (MDR) pathogens (12–14) with potentially life-threatening complications (30% vs. 10% of mortality in cases of carbapenem resistance) (15) and a higher recurrence risk (13).

Surgical complications after KT were associated with AGPN but results were mixed and thus inconclusive (16); some authors suggested that there is greater AGPN incidence among patients who experienced ureteral stenosis (UrS) (17).

It can be affirmed that all these characteristics, especially the impact on graft function, may occur and evolve differently in elderly or extended criteria donors (ECDs), but there is limited case evidence reported in the literature.

Identifying phenotypes and determinants for AGPN may be particularly important for KTs, where inappropriate antibiotic therapy may pose crucial problems with immunosuppressive medication and cause MDR pathogen selection (13, 14).

Our study aimed to retrospectively analyze our cohort of consecutive KTs with many elderly donors, evaluating the clinical and microbiological characteristics of all AGPN episodes and considering the impact of AGPN on allograft function and survival.

## 2 Methods

### 2.1 Study patients and ethical statement

We performed a retrospective observational study of all consecutive adult recipients who received a KT at Turin University Renal Transplant Center “A. Vercellone” from January 2011 to December 2018. The local Ethical Committee approved this study (*Comitato Etico Interaziendale A.O.U. Città Della Salute e Della Scienza di Torino - A.O. Ordine Mauriziano - A.S.L. Città di Torino*, resolution number 1449/2019 on 11 August 2019). This study was conducted according to the principles of the Helsinki and Istanbul Declarations. All participants provided written informed consent about the use of their data/information for this retrospective analysis. Follow-up was terminated on 31 December 2019.

### 2.2 Exposure

According to the *American Society of Transplantation Infectious Diseases Community of Practice* indications (5), AGPN was clinically suspected when suggestive clinical symptoms (i.e., fever with flank/allograft pain and/or symptoms of lower UTI including frequency, urgency, dysuria, and/or suprapubic pain) and typical laboratory findings (i.e., urinalysis showing leukocyte counts >10 per mm^3^ or > 10^4^ colony-forming units of bacteria per milliliter of urine; leukocytosis either with or without bacteria isolated from blood cultures) appeared. Additionally, each clinically suspected episode was further investigated with magnetic resonance imaging (MRI) within 24 h of initial symptoms for radiological confirmation/exclusion [detailed protocol is described in Faletti et al. (18)].

AGPN episodes that required hospitalization in different centers were considered and collected in case of available MRI confirmation. All of the AGPN episodes were evaluated by three authors (RT, GC, and AM) through a retrospective review of hospital records. AGPN episodes were then classified according to radiological characteristics (multifocal vs. unifocal and abscessed vs. non-abscessed) and time after transplantation [early (<3 months after KT) vs. late (3–12 months)].

### 2.3 Posttransplant management and data collection

All patients were initially managed by the Renal Transplant Center (Hub center) and received induction therapy (steroids and basiliximab/anti-thymocyte globulin [ATG] according to donor type and immune risk) and maintenance immunosuppression mainly composed of tacrolimus (10–15 ng/mL for the first 3 months and 6–8 ng/mL thereafter), mycophenolate mofetil/mycophenolic acid, and/or steroids (progressively tapered to 5 mg/day). The urological anastomosis was usually performed with the Lich-Gregoire antireflux technique and intraoperative double-J ureteral stenting (removed 4 weeks post-KT); the transurethral bladder catheter was usually maintained for 3–5 days.

After discharge, post-transplant care followed a standardized schedule, and every recipient was followed by the transplant center (Hub center) with at least 1 annual visit and by the local nephrologist (11 peripherical centers covering most of the Piedmont region) for their periodical follow-up.

All clinical and medical information (including donor data and immunosuppressive medications) was collected from patients’ charts. Renal allograft function (eGFR) was estimated by the Chronic Kidney Disease Epidemiology Collaboration (CKD-EPI) equation. We included eGFR values at discharge after transplantation and first year after transplant, considering a period after AGPN episodes of at least 2 weeks and with an eGFR documented stabilization in >2 tests in the absence of AGPN-induced acute kidney injury.

### 2.4 Outcomes

The primary purpose of this study was to evaluate the effect of AGPN on death-censored graft survival, stratifying for donor age to assess the potential impact of elderly donors.

Secondary purposes included identifying risk factors for AGPN, the impact of AGPN on patient survival rates, the possible modification in eGFR (available at discharge and first year after transplant), and the potential differences according to radiological presentation.

We subsequently compared death-censored graft survival rates and eGFR between KTR with and without AGPN. To discriminate at least the potential impact of donors in determining AGPN, we also investigated the AGPN rate in patients who received kidneys from the same donor (paired grafts).

### 2.5 Statistical methods

The distribution of continuous variables, overall and for subgroups, was analyzed with the Kolmogorov–Smirnov test. Based on their non-Gaussian distribution, we described age, eGFR, and follow-up with median and interquartile range (IQR).

Between-group comparisons of continuous variables were performed with the non-parametric Mann–Whitney test. To assess the effect of AGPN on the post-transplantation evolution of the eGFR, we compared eGFR at the first year vs. at discharge with the Wilcoxon signed-rank test.

To model the value of eGFR at the first year for patients with AGPN, we used linear regression with variables of interest with potential impact on AGPN severity (induction with ATG, multifocal presentation, abscedation, and donor age) as predictors. Considering the characteristics of the dependent variable, we used the *ln*-transformed eGFR at the first year to improve the accuracy of the linear regression model.

Categorical variables are presented as fractions, and Pearson’s r, for small samples. Fisher’s exact test was used to compare groups. The odds ratios (ORs) with a 95% confidence interval were used to measure relative risk.

Univariate survival analysis was performed utilizing the Kaplan–Meier method with the log-rank test to compare strata. The significance level for all tests was set at an *α*-value of <0.05.

Statistical analysis was performed with IBM SPSS Statistics for Windows, version 28.0.1*α* (IBM Corp., Armonk, NY, USA).

## 3 Results

### 3.1 Population characteristics

We analyzed 939 consecutive KTs, including 224 patients who received kidneys from the same donor (paired grafts). Among this population, 130 AGPN episodes in the first year after transplant were recorded based on the clinical criteria (5), but 21 of them (16.2%) were not confirmed by MRI and were analyzed separately.

Patient and donor characteristics stratified for AGPN occurrence are reported in Table 1.

**Table 1:** Characteristics of the studied population according to AGPN occurrence.

	AGPN (*n* = 109)	non-AGPN (*n* = 788)	*p*
Women, *n* (%)	38 (34.9)	280 (35.5)	0.915
Age at KT, years (IQR)	63 (50–71)	55 (46–65)	0.945
Age ≥ 65, years (%)	30 (27.5)	198 (25.1)	0.639
Donor age, years (IQR)	63 (50–71)	60 (48–71)	0.347
Extended-Criteria Donors^a^, *n* (%)	45 (41.3)	345 (43.8)	0.680
Previous KT, *n* (%)	21 (19.3)	101 (12.8)	0.074
Living donor, *n* (%)	10 (9.2)	56 (7.1)	0.434
Dual kidney transplantation, *n* (%)	4 (3.7)	24 (3)	0.766
Acute rejection episodes during the first year, *n* (%)	15 (13.8)	87 (11.3)	0.426
Induction immunosuppressive therapy			
ATG, *n* (%)	30 (27.5)	202 (25.6)	0.726
Basiliximab, *n* (%)	81 (72.5)	604 (74.4)	0.630
Ureteral Stenosis on KT, *n* (%)	17 (15.6)	48 (6.1)	**0.001**
UTI episodes			
Urinalysis with CFU of bacteria >10^6^/ml, *n* (%)	38 (34.9)	107 (13.7)	<0.005
Recurrent urinalyses with positive urine culture, *n* (%)	27 (71.1)^b^	59 (55.1)^b^	0.063
Identification of MDR bacteria on positive urine culture, *n* (%)	14^c^ (36.8)	18^d^ (16.8)	**0.022**
AGPN Clinical Characteristics			
Transfer in the ICU, *n* (%)	3 (2.8)	/	
Use of vasopressors, *n* (%)	1 (0.9)	/	
Early/late, *n* (%)	87 (79.8) / 21 (19.3)	/	/
Solitary/recurrent, *n* (%)	88 (80.7) / 21 (19.3)	/	/
AGPN MRI Characteristics			
Multifocal/unifocal, *n* (%)	73 (67.0) / 36 (33.0)	/	/
Abscessed/not abscessed, *n* (%)	22 (20.2) / 87 (79.8)	/	/

AGPN, acute graft pyelonephritis; KT, kidney transplant; IQR, interquartile range; ATG, anti-thymocyte globulin; UTI, urinary tract infection; MDR, multidrug resistance; ICU, intensive care unit; MRI, magnetic resonance imaging.

^a^ According to Crystal City criteria.

^b^ Percentage of the total number of urinalysis with CFU of bacteria > 10^6^/ml in each group.

^c^ K. pneumoniae carbapenemase producing in 4 of 14, ESBL-producing E. coli in 7 of 14, MDR P. aeruginosa in 1 of 14, vancomycin-resistant Enterococcus faecium in 1 of 14, and others in 1 of 14.

^d^ K. pneumoniae carbapenemase producing in 8 of 18, ESBL-producing E. coli in 6 of 18, vancomycin-resistant Enterococcus faecium in 2 of 21, and others in 2 of 21. Bold values denote statistical significance at the *p* < 0.05 level.

Both groups have similar profiles, considering gender, patient and donor age, induction therapies, and rejection episodes. Worthy of mention, donor age was similar between groups (63 years [50–71] in AGPN and 60 [48–71] in non-AGPN, *p* = 0.347), with 449 of 897 (50.1%) KTs with a donor >60 years. Furthermore, ECDs [defined according to the Cristal City criteria (19, 20)] are equally distributed in both groups (41.3% in AGPN vs. 43.8% in non-AGPN, *p* = 0.680), reflecting our significant utilization with a preferential old-for-old allocation (21).

Patients in the AGPN group showed, as expected, a high percentage of positive urine culture (34.9% vs. 15.7%, *p* < 0.005), a high number of UTIs due to MDR bacteria (36.8% vs. 16.8%, *p* = 0.022), and a trend toward more recurrent episodes (71.1% of total positive urine cultures in the AGPN group vs. 55.1% in non-AGPN, *p* = 0.063).

Among potential risk factors, some patients experienced AGPN before double-J removal (20 of 108, 18.3%), but only UrS confirmed by antegrade pyelography appears to be significantly associated with AGPN (OR 2.9 [CI 95% 1.6 to 5.2]; *p* = 0.001).

### 3.2 Association between AGPN, patient and kidney survival, and graft function

**Figure 1:**
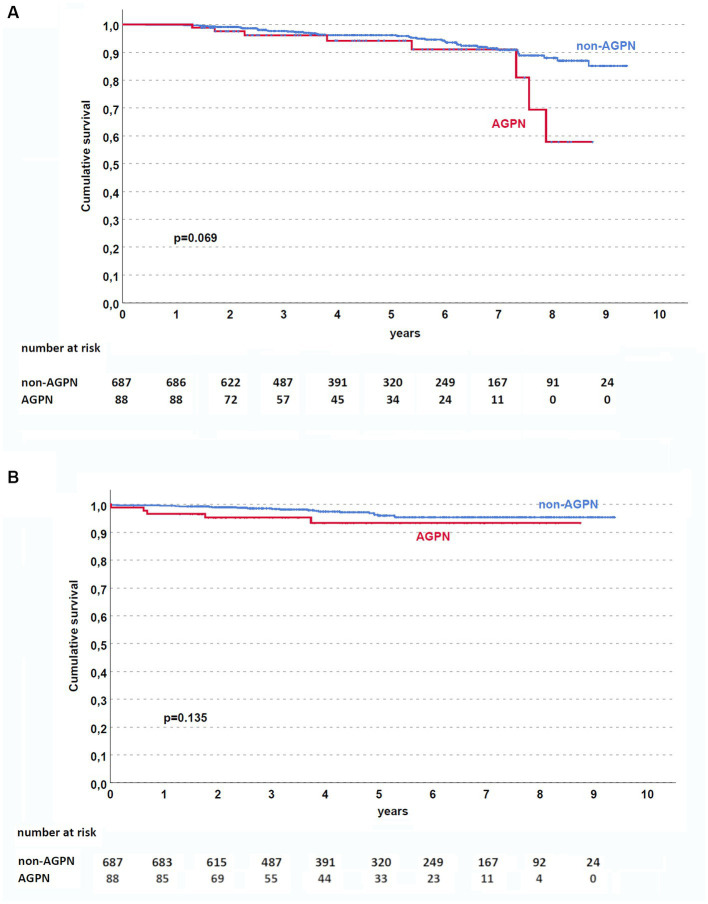
Kaplan–Meier curves in the studied population. AGPN and non-AGPN (excluding retransplant) had a similar patient **(A)** and death-censored graft **(B)** survival. AGPN, acute graft pyelonephritis.

**Figure 2:**
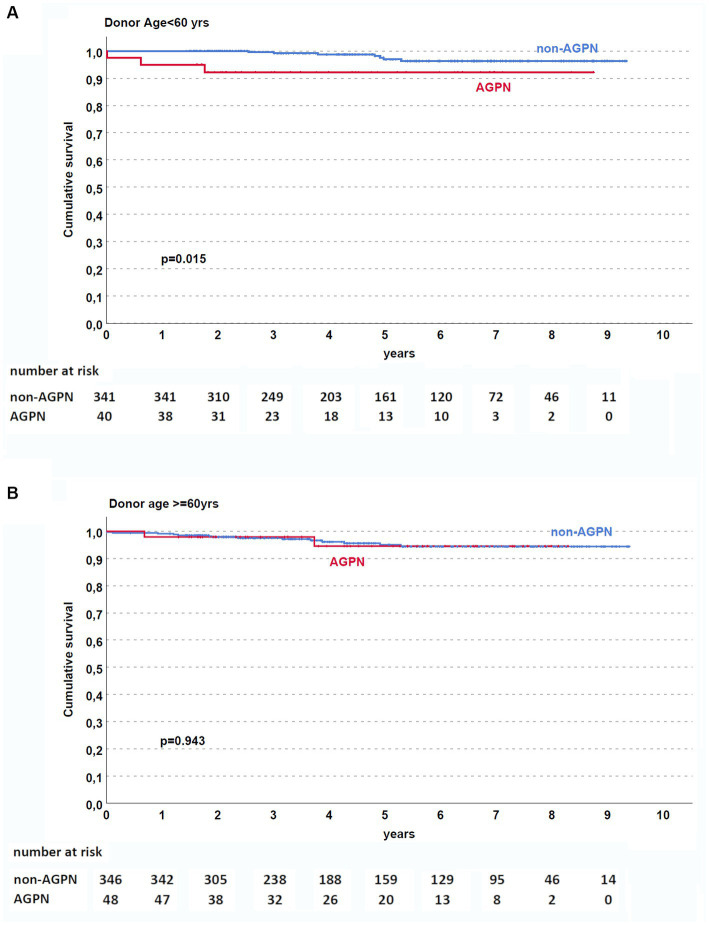
Kaplan–Meier curves according to donor age [**(A)** <60 years and **(B)** ≥ 60 years]. In patients (excluding retransplant) with donors <60 years, AGPN was associated with reduced death-censored graft survival. AGPN, acute graft pyelonephritis.

Although AGPN has no apparent effect on both patient and death-censored kidney survival in the entire population (Figures 1A,B, respectively), KT patients who experienced AGPN with a donor age < 60 years had low death-censored graft survival (Figure 2).

**Figure 3:**
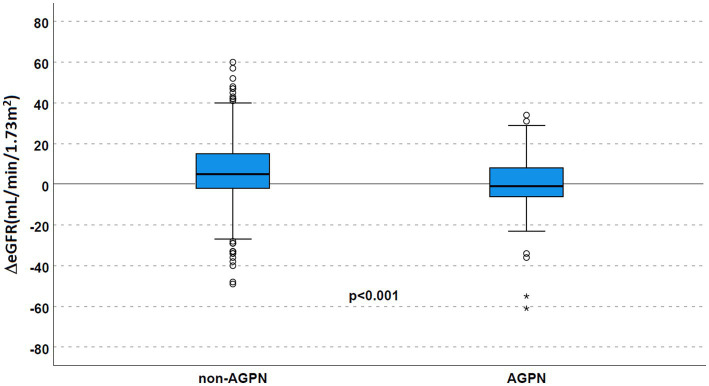
Renal function according to AGPN occurrence. ΔeGFR was reduced 1 year after transplant in patients who experienced AGPN. AGPN, acute graft pyelonephritis; eGFR: estimated glomerular filtration rate.

Despite similar kidney function after transplant, AGPN was associated with a lower eGFR at the first year (median eGFR 40 mL/min/1.73 m^2^ in the AGPN group vs. 52 mL/min/1.73 m^2^, *p* < 0.001 with a ΔeGFR 5 mL/min/1.73 m^2^ [−2–15] in non-AGPN vs. −1 [−6.5–8.5], *p* < 0.001, Figure 3).

**Figure 4:**
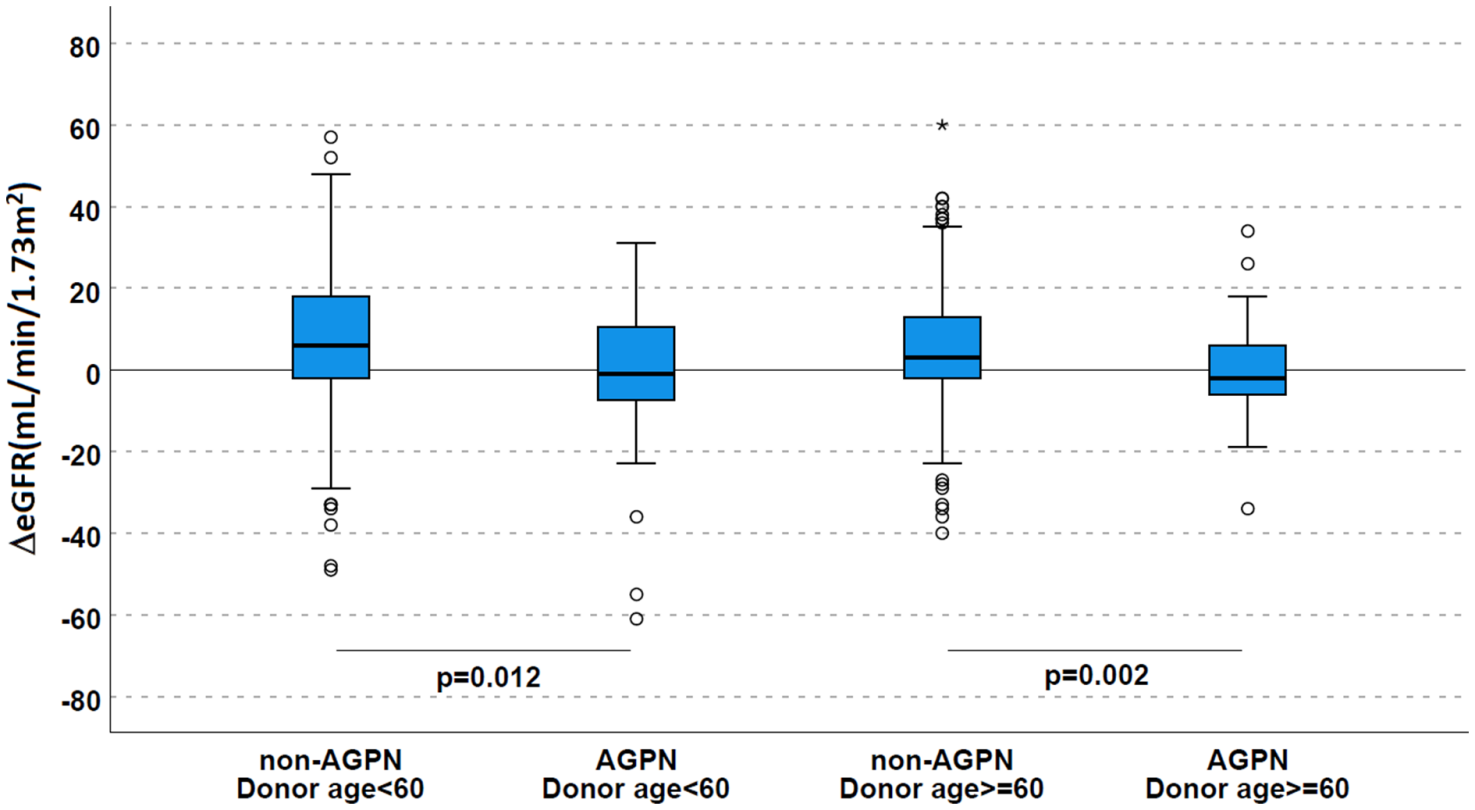
Renal function according to AGPN occurrence and donor age. ΔeGFR was reduced 1 year after transplant in patients who experienced AGPN, irrespective of donor age. AGPN, acute graft pyelonephritis; eGFR, estimated glomerular filtration rate.

Stratifying for donor age (Table 2 and Figure 4), this trend toward a significantly reduced allograft function was confirmed (ΔeGFR 6 mL/min/1.73 m^2^ [−2–18] in non-AGPN vs. –1 [−8–11] in KTs with donors <60 years and 3 [−2–13] vs. −2 [−6–6.25] in donors ≥60 years).

**Table 2:** ΔeGFR at transplant and the 1-year f/up in the studied population according to the AGPN occurrence.

	AGPN (*n* = 109)	non-AGPN (*n* = 809)	*p*
eGFR at transplant, mL/min/1.73m^2^	36 (26–61)	42 (32–59)	0.06
eGFR first year after KT, mL/min/1.73m^2^	40 (26–60.5)	52 (38–67)	**< 0.001**
ΔeGFR (overall population), mL/min/1.73m^2^	–0.2 [−6.5–8.5]	6 [−2–15]	**< 0.001**
Donor age < 60 years	–1 [−8–11]	6 [−2–18]	**0.012**
Donor age ≥ 60 years	–2 [−6–6.25]	3 [−2–13]	**0.002**

eGFR, estimated glomerular filtration rate; AGPN, acute graft pyelonephritis. Bold values denote statistical significance at the *p* < 0.05 level.

### 3.3 Differences in AGPN groups stratified for clinical and radiological characteristics

AGPN episodes were therefore stratified according to donor age, clinical features (early [< 3 months post-KT] or late [3–12 months], solitary/recurrent), and, based on MRI evaluation, multifocal/unifocal, and with/without abscedation.

Early, solitary, multifocal, and non-abscessed AGPN cases were prevalent in our cohort (Table 1). No significant difference was observed in early vs. late, solitary vs. recurrent (apart from increased evidence of recurrent positive urine culture in recurrent AGPN), or in AGPN with or without abscedation (Supplementary Tables S1–S3).

The multifocal pattern demonstrates a different profile: KT patients who experienced a multifocal AGPN show a trend toward a younger age at transplant, received more frequent ATG at induction, and showed a lower donor age. Interestingly, despite a better eGFR at transplant, justified by the difference in donor ages, the eGFR tends to overlap among groups at first year (Supplementary Table S4).

Based on the differences observed in graft survival, we stratified our population for donor age classes (<60 and ≥ 60 years): AGPN with donors <60 years, apart from a younger age at KT (48 years [40–55] vs. 63 [55–69]), have similar characteristics (including gender, urinalysis with positive urine culture, and UrS) but have preferentially received ATG induction (40.4% vs. 17.7%, *p* < 0.001) and, interestingly, have more frequently experienced multifocal pattern (73.9% vs. 56.7%, *p* = 0.026) or abscedation (28.3% vs. 11.7%, *p* = 0.074) as detected in the MRI.

Considering that ATG induction has been associated with BK polyomavirus infection, which can increase the risk of graft damage and loss, we assess the number of BK virus nephritis (BKVN) in patients who lost their grafts (n = 30): three of them experienced BKN with similar distribution in AGPN and non-AGPN (1 of 7 [14.3%] vs. 2 of 23 [8.7%], respectively, *p* = 0.564).

We assessed a multiple linear regression model to better evaluate the impact of specific conditions on 1-year eGFR in patients with AGPN. A first analysis highlighted an asymmetric distribution of the residuals, violating the assumption of normality for linear regression. We, therefore, decided to use a logarithmic transformation of the response variable to improve our concerns.

Through linear regression, with a multifocal presentation, presence of abscedation, donor age > 60 years, and ATG at induction as predictors, we identified that the model explained more than 75% of the variation in log(eGFR) (*R*^2^ = 0.811). The *F* statistic resulted significantly (*p* < 0.001), indicating that the model predicted eGFR at the first year better than the mean. All coefficients were significant, confirming that these variables contribute to the model. In particular, a multifocal presentation contributes more than donor age, ATG, and evidence of abscedation (Table 3).

**Table 3:** Linear regression analysis for a significant determinant of *ln*(eGFR) at first year.

	*B*	Standard error	Beta coefficient	*p*
Donor age ≥ 60 years vs. <60 years	1.791	0.248	0.364	**<0.001**
Multifocal presentation (yes vs. no)	2.211	0.262	0.487	**<0.001**
AGPN with abscedation (yes vs. no)	1.237	0.376	0.150	**0.001**
ATG at induction (yes vs. no)	1.169	0.350	0.165	**0.001**

AGPN, acute graft pyelonephritis; ATG, anti-thymocyte globulin. Bold values denote statistical significance at the *p* < 0.05 level.

To discriminate at least the potential impact of donors in determining AGPN, we also investigated the AGPN rate in paired kidneys. The absence of agreement (51 patients experienced AGPN but only in 4 cases did it occur in both recipients, Cohen’s *K* coefficient = 0.116), also in early AGPN (45 episodes but only 3 in paired grafts, *K* = 0.102), suggests the lack of a donor effect in AGPN occurrence.

### 3.4 Characteristics of patients with a potential clinical diagnosis but without radiological signs of AGPN

As previously reported, 21 out of 130 patients with clinically-based AGPN showed no radiological signs of kidney involvement based on the MRI.

These patients had not developed severe infections or sepsis, had similar characteristics considering all examined previous variables but had significant evidence of urinalyses with positive urine cultures and, despite a slight increase during the first year, showed a reduced eGFR vs. the non-AGPN group (Supplementary Table S5).

## 4 Discussion

AGPN is one of the most frequent infections in KTs. In the past few years, this pathological process has been considered a relatively “benign” condition (22, 23). More recently, an increasing number of papers have highlighted the potential role of AGPN in determining reduced graft function and kidney survival (6, 7, 24–27).

One potential limitation of all previous studies in this field is the adoption of clinical criteria alone for AGPN diagnosis. However, as previously reported, especially regarding native kidneys, diffusion-weighted MRI with an apparent diffusion coefficient seems to be a reliable diagnostic tool with a very low false negative rate (18, 28, 29).

In our experience, MRI confirmation allows us to identify a significant percentage of patients with no parenchymal signs of infection (≈15%) and thus should be duly considered. On the one hand, this observation could be important in the transplant setting, where antibiotic overtreatment may favor MDR pathogen selection and rejection risk due to the potential minimization of immunosuppressive therapy after diagnosis. On the other hand, the analysis of the subgroup of KTs with clinical signs of AGPN and a negative MRI revealed a suboptimal kidney function associated with recurrent UTIs, suggesting, as previously described, a potential negative impact of UTIs by themselves on graft outcome and the need for continued surveillance of these patients (9, 10, 30).

Positive urinalyses and MDR detection are more common in AGPN, and this was the case in our population. At the same time, empiric and inappropriate antibiotic therapy is associated with a higher risk of bacteriemia due to MDR in SOTs (5, 13, 31), further corroborating our approach.

Many conditions that involve the urological tract are associated with UTIs and AGPN (e.g., bladder dysfunction, vesicoureteral reflux, and diabetes), although their impact has been debated (32–36). Our analysis identifies UrS as a significant risk factor for radiologically confirmed AGPN.

Similarly, Karam et al. highlighted a considerable incidence of AGPN among patients with UrS (29% vs. 14.4% in KTs without UrS, *p* < 0.05) (17). UrS requiring a surgical approach determines a higher risk of AGPN, primarily when hydronephrosis is associated (17, 37). UrS and/or ureteral necrosis can lead to urine leakage into the abdomen and easier urinary tract contamination from intestinal bacteria. Besides, the surgical approach always represents a potential infectious risk, even more so among the immunosuppressed population.

An important topic of our study is the specific correlation of AGPN with eGFR post-KT. We found that patients with AGPN experienced a reduction in eGFR at the first year, irrespective of donor age. However, this condition determined an inferior death-censored graft survival in patients with donors <60 years; this subgroup concurrently experienced more frequent multifocal presentation and abscedation and preferentially received ATG induction. Although the correlation between high immunosuppression (i.e., after acute rejection episodes) and increased infection (including AGPN) rates is well established (8), the characteristics of different populations, especially with elderly donors, have not been intensively investigated.

Recent studies that evaluated the impact of AGPN on eGFR showed negative effects on graft survival and eGFR, but in populations with a limited number of old recipients/donors [recipients and donor age 52.6 [40.15–60.7] and 53 [41–62] in Maanaoui et al. (6), and 51.0 ± 14.1 and 52.1 ± 16.4 in Pacaud et al. (7)]. Additionally, neither of them routinely prescribed radiological confirmation for an AGPN diagnosis. Our population reflects our allocation policy with a homogenous clinical and therapeutical approach (20, 21), probably emphasizing the niche of KTs that developed increased organ damage and impairment of reserve graft function after “severe” AGPN (abscessed or multifocal). This consideration is highlighted by the multivariate linear regression model, where multifocal presentation (with a strong coefficient), donor age, abscedation, and ATG induction are independent predictors of eGFR at the first year. These data, combined with the evidence that multifocal presentation is more common among patients with ATG induction and young age, suggest that these KTs could be more susceptible to this severe presentation and that patients with multifocal features could be treated more intensively and actively monitored to reduce the potential impact on eGFR. Since abscedation and multifocal presentation are unrelated and abscedation does not seem to be influenced by induction therapy, this pattern could depend more on local conditions (i.e., the specific pathogen involved).

Our study has some limitations (retrospective design, absence of routine post-AGPN protocol biopsies, and availability of limited eGFR time-points). We are also aware that MRI assessment is expensive, and its availability broadly differs among centers. However, we suggest that it offers some advantages over CT scans (especially for radiation exposure) and better reproducibility than contrast-enhanced ultrasonography, also depicting some patterns (multifocal involvement/evidence of abscedation) that may be related to adverse outcomes requiring careful management with eventually prolonged therapy and surveillance.

Additionally, our real-life analysis of a population of recipients with a significant percentage of elderly recipients/donors may have identified a niche group of KTs requiring prompt and effective therapy to respond to AGPN episodes and avoid renal scarring development and long-term allograft dysfunction.

## 5 Conclusion

AGPN, influenced by multifocal presentation, ATG induction, donor age, and abscedation, affects kidney function and significantly impacts allograft survival in KTs with donors <60 years.

Although we are aware of limited availability and costs, radiological confirmation may help in this setting to establish the appropriate antibiotic therapy, avoid overtreatment, and prevent the potential risk of allograft dysfunction.

## Data availability statement

The raw data supporting the conclusions of this article will be made available by the authors, without undue reservation.

## Ethics statement

The studies involving humans were approved by Comitato Etico Interaziendale A.O.U. Città Della Salute e Della Scienza di Torino - A.O. Ordine Mauriziano - A.S.L. Città di Torino. The studies were conducted in accordance with the local legislation and institutional requirements. The participants provided their written informed consent to participate in this study.

## Author contributions

RT: Conceptualization, Formal analysis, Writing – original draft. GC: Formal analysis, Investigation, Writing – original draft. AM: Data curation, Formal analysis, Methodology, Validation, Visualization, Writing – review & editing. GA: Writing – review & editing, Formal analysis. FF: Writing – review & editing, Data curation, Methodology, Software, Visualization. CD: Supervision, Writing – review & editing. EG: Supervision, Writing – review & editing. MV: Supervision, Writing – review & editing. RF: Supervision, Writing – review & editing, Investigation, Methodology. AB: Supervision, Writing – review & editing. PG: Supervision, Writing – review & editing. CC: Supervision, Writing – review & editing. RC: Supervision, Writing – review & editing. FM: Supervision, Writing – review & editing, Formal analysis. SC: Supervision, Writing – review & editing. FR: Supervision, Writing – review & editing. PF: Supervision, Writing – review & editing. LB: Conceptualization, Data curation, Formal analysis, Investigation, Methodology, Project administration, Resources, Supervision, Validation, Writing – review & editing.
