# Supplementary material for: Different profiles of acute graft pyelonephritis among kidney recipients from standard or elderly donors
Source: Front Med (Lausanne). 2024 May 14;11:1342992. doi: 10.3389/fmed.2024.1342992 (PMC11130444; doi:10.3389/fmed.2024.1342992)
Supplement: Supplementary file 1 [file Data_Sheet_1.pdf]

Supplementary Material

Table S1. Characteristics of the AGPN group according to the presentation (early vs. late).

	Early AGPN (n=89)	Late AGPN (n=21)	p
Women, n (%)	32 (36)	5 (23.8)	0.314
Age at KT, yrs (IQR)	56 (46-65)	50 (38-66)	0.426
Age ≥ 65, yrs (%)	23 (25.8)	7 (33.3)	0.590
Donor age, yrs (IQR)	63 (52-72)	62 (45.5-69)	0.404
Induction immunosuppressive therapy			
ATG, n (%)	25 (28)	4 (19)	0.426
Basiliximab, n (%)	64 (71.9)	17 (81)	0.584
Ureteral Stenosis on KT, n (%)	14 (15.7)	3 (14.3)	1.00
UTI episodes			
Urinalysis with CFU of bacteria $>10^6$/ml, n (%)	29 (32.6)	8 (38.1)	0.799
Recurrent urinalyses with positive urine culture, n (%)	19 (65.5)*	7 (87.5)*	0.391
Identification of MDR bacteria on positive urine culture, n (%)	11 (37.9)*	3 (37.5)*	1.00
Abscessed on MRI, n (%)	19 (21.3)	3 (14.3)	0.556
Multifocal on MRI, n (%)	59 (66.3)	14 (66.7)	1.00
Solitary, n (%)	72 (80.9)	18 (85.7)	1.00
eGFR at transplant, mL/min/1.73m^2	35 (25-61)	36 (31.5-60)	0.417
eGFR one year after KT, mL/min/1.73m^2	40 (27-61)	42 (23.5-56.5)	0.611

AGPN: acute graft pyelonephritis; KT: kidney transplant; IQR: interquartile range; ATG: anti-thymocyte globulin; UTI: urinary tract infection; MDR: multi-drug resistance; MRI: Magnetic Resonance Imaging; eGFR: estimated glomerular filtration rate

*Percentage of the total number of urinalysis with CFU of bacteria $>10^6$/ml in each group

Table S2. Characteristics of the AGPN group according to the presentation (solitary vs. recurrent).

	Solitary AGPN (n=90)	Recurrent AGPN (n=18)	p
Women, n (%)	33 (36.3)	5 (27.8)	0.594
Age at KT, yrs (IQR)	56 (44-65)	56.5 (40.25-67.25)	0.613
Age $\geq$ 65, yrs (%)	24 (26.4)	6 (33.3)	0.570
Donor age, yrs (IQR)	62 (49-71)	64 (52.25-72.5)	0.616
Induction immunosuppressive therapy			
ATG, n (%)	26 (28.6)	4 (22.2)	0.775
Basiliximab, n (%)	66 (72.5)	15 (83.3)	0.555
Ureteral Stenosis on KT, n (%)	15 (16.5)	2 (11.1)	0.733
UTI episodes			
Urinalysis with CFU of bacteria $>10^6$/ml, n (%)	29 (32.2)	9 (50)	0.178
Recurrent urinalyses with positive urine culture, n (%)	18 (62.1)*	9 (100)*	**0.038**
Identification of MDR bacteria on positive urine culture, n (%)	12 (41.4)*	2 (22.2)*	0.438
Abscessed on MRI, n (%)	20 (22.0)	2 (11.1)	0.520
Multifocal on MRI, n (%)	58 (63.7)	15 (83.3)	0.086
Early (<3 months post-KT), n (%)	72 (80)	15 (83.3)	1.00
eGFR at transplant, mL/min/1.73m^2	35 (26-61)	40.5 (26.5-60)	0.735
eGFR one year after KT, mL/min/1.73m^2	40 (26-60)	37.5 (25-71.25)	1.00

AGPN: acute graft pyelonephritis; KT: kidney transplant; IQR: interquartile range; ATG: anti-thymocyte globulin; UTI: urinary tract infection; MDR: multi-drug resistance; MRI: Magnetic Resonance Imaging; eGFR: estimated glomerular filtration rate

*Percentage of the total number of urinalysis with CFU of bacteria >10^6/ml in each group

Table S3. Characteristics of the AGPN group according to the radiological presentation (abscessed vs. not abscessed).

	Abscessed AGPN (n=22)	Non-abscessed AGPN (n=87)	p
Women, n (%)	10 (45.5)	28 (32.2)	0.317
Age at KT, yrs (IQR)	53.5 (45.5-61.75)	56 (44-66)	0.523
Age ≥ 65, yrs (%)	3 (13.6)	27 (31)	0.082
Donor age, yrs (IQR)	58.5 (45-69)	63 (52-72)	0.173
Induction immunosuppressive therapy			
ATG, n (%)	6 (27.3)	24 (27.6)	1.00
Basiliximab, n (%)	17 (77.3)	64 (73.6)	0.792
Ureteral Stenosis on KT, n (%)	3 (13.6)	14 (16.1)	1.00
UTI episodes			
Urinalysis with CFU of bacteria >10^6/ml, n (%)	10 (45.5)	28 (32.2)	0.317
Recurrent urinalyses with positive urine culture, n (%)	8 (80)*	19 (67.9)*	0.690
Solitary, n (%)	20 (90.9)	71 (81.6)	0.520
eGFR at transplant, mL/min/1.73m^2	31 (24.75-49.75)	36 (26-62)	0.270
eGFR one year after KT, mL/min/1.73m^2	43.5 (31.5-58.75)	40 (25-61)	0.695

AGPN: acute graft pyelonephritis; KT: kidney transplant; IQR: interquartile range; ATG: anti-thymocyte globulin; UTI: urinary tract infection; MDR: multi-drug resistance; MRI: Magnetic Resonance Imaging; eGFR: estimated glomerular filtration rate

*Percentage of the total number of urinalysis with CFU of bacteria >10^6/ml in each group

Table S4. Characteristics of the AGPN group according to the radiological presentation (multifocal vs. unifocal involvement).

	**Multifocal AGPN (n=73)**	**Unifocal AGPN (n=36)**	**p**
Women, n (%)	24 (32.9)	14 (38.9)	0.669
Age at KT, yrs (IQR)	53 (43-64)	57.5 (48-67)	0.177
Age ≥ 65, yrs (%)	16 (21.9)	14 (38.9)	**0.052**
Donor age, yrs (IQR)	59 (47-67.5)	69 (59-74.75)	**0.003**
Induction immunosuppressive therapy			
ATG, n (%)	24 (32.9)	6 (16.7)	**0.057**
Basiliximab, n (%)	50 (68.5)	31 (86.1)	**0.037**
Ureteral Stenosis on KT, n (%)	15 (20.5)	2 (5.6)	**0.034**
UTI episodes			
Urinalysis with CFU of bacteria $>10^6$/ml, n (%)	26 (35.6)	12 (33.3)	1.00
Recurrent urinalyses with positive urine culture, n (%)	19 (73.1)*	8 (66.7)*	0.714
Abscessed on MRI, n (%)	14 (19.2)	8 (22.2)	0.801
Solitary, n (%)	58 (79.5)	33 (91.7)	0.086
eGFR at transplant, mL/min/1.73m^2	42 (27-67.5)	30 (24.25-39)	**0.003**
eGFR one year after KT, mL/min/1.73m^2	44 (30-67.5)	33 (25-50)	**0.054**

AGPN: acute graft pyelonephritis; KT: kidney transplant; IQR: interquartile range; ATG: anti-thymocyte globulin; UTI: urinary tract infection; MDR: multi-drug resistance; MRI: Magnetic Resonance Imaging; eGFR: estimated glomerular filtration rate

*Percentage of the total number of urinalysis with CFU of bacteria $>10^6$/ml in each group

Table S5. Characteristics of suspicious AGPN (not confirmed by MRI) vs. non-AGPN.

	Non AGPN (n=788)	Suspicious AGPN (n=21)	p
Women, n (%)	280 (35.5)	10 (47.6)	0.258
Age at KT, yrs (IQR)	55 (46-65)	58 (48.5-64)	
Age ≥ 65, yrs (%)	198 (25.1)	4 (19)	0.619
Donor age, yrs (IQR)	60 (48-71)	63 (53-70)	
Induction immunosuppressive therapy			
ATG, n (%)	202 (25.6)	5 (23.8)	1.00
Ureteral Stenosis on KT, n (%)	48 (6.1)	2 (9.5)	0.377
UTI episodes			
Urinalysis with CFU of bacteria $>10^6$/ml, n (%)	107 (13.7)	20 (95.2)	**<0.001**
UTI due to MDR bacteria (%)	18* (16.8)	3 *(15)	0.765
eGFR at transplant, mL/min/1.73m^2	42 (32-59)	38 (30.5-53.5)	0.497
eGFR one year after KT, mL/min/1.73m^2	52 (38-67)	40 (27.5-52.5)	**0.009**

AGPN: acute graft pyelonephritis; KT: kidney transplant; IQR: interquartile range; ATG: anti-thymocyte globulin; UTI: urinary tract infection; MDR: multi-drug resistance; MRI: Magnetic Resonance Imaging; eGFR: estimated glomerular filtration rate

*Percentage of the total number of urinalysis with CFU of bacteria $>10^6$/ml in each group
